# Gapless reference genome assembly of *Didymella glomerata*, a new fungal pathogen of maize causing Didymella leaf blight

**DOI:** 10.3389/fpls.2022.1022819

**Published:** 2022-10-26

**Authors:** Wendi Ma, Jun Yang, Junqiang Ding, Wensheng Zhao, You-Liang Peng, Vijai Bhadauria

**Affiliations:** ^1^ Department of Plant Pathology, College of Plant Protection, China Agricultural University, Beijing, China; ^2^ Ministry of Agriculture and Rural Affairs, Key Laboratory for Crop Pest Monitoring and Green Control, China Agricultural University, Beijing, China; ^3^ College of Agronomy, Henan Agricultural University, Zhengzhou, China

**Keywords:** emerging maize diseases, fungal genomes, effectors, CAZymes, secondary, metabolism genes, genetic source of Didymella leaf blight resistance

## Abstract

Didymella leaf blight (DLB) caused by *Didymella glomerata* is a new fungal disease of maize (*Zea mays*), first detected in 2021 in Panjin, Liaoning province of China. Here we report the reference genome assembly of *D. glomerata* to unravel how the fungal pathogen controls its virulence on maize at the molecular level. A maize-infecting strain Pj-2 of the pathogen was sequenced on the Illumina NovaSeq 6000 and PacBio Sequel II platforms at a 575-fold genomic coverage. The 33.17 Mb gapless genome assembly comprises 32 scaffolds with L/N_50_ of 11/1.36 Mb, four of which represent full-length chromosomes. The Pj-2 genome is predicted to contain 10,334 protein-coding genes, of which 211, 12 and 134 encode effector candidates, secondary metabolite backbone-forming enzymes and CAZymes, respectively. Some of these genes are potentially implicated in niche adaptation and expansion, such as colonizing new hosts like maize. Phylogenomic analysis of eight strains of six *Didymella* spp., including three sequenced strains of *D. glomerata*, reveals that the maize (Pj-2)- and *Chrysanthemum* (CBS 528.66)-infecting strains of *D. glomerata* are genetically similar (sharing 92.37% genome with 98.89% identity), whereas Pj-2 shows truncated collinearity with extensive chromosomal rearrangements with the *Malus*-infecting strain M27-16 of *D. glomerata* (sharing only 55.01% genome with 88.20% identity). Pj-2 and CBS 528.66 carry four major reciprocal translocations in their genomes, which may enable them to colonize the different hosts. Furthermore, germplasm screening against Pj-2 led to the identification of three sources of DLB resistance in maize, including a tropical inbred line CML496. DLB resistance in the line is attributed to the accumulation of ROS H_2_O_2_ in the apoplastic space of the infected cells, which likely restricts the fungal growth and proliferation.

## Introduction

*Didymella* (syn. *Peyronellaea* or *Phoma*) *glomerata* (Corda) Q. Chen & L. Cai is an ascomycete fungal pathogen, which causes Didymella leaf blight (DLB). The pathogen has a broad host range and is known to infect both monocots and dicots, including although not limited to flax (*Linum usitatissimum*) (Reeder and Vanterpool, 1953), oilseed rape (*Brassica napus*) (Taber and Vanterpool, 1963), bread wheat (*Triticum aestivum*), durum wheat (*Triticum turgidum*) and triticale (*Triticale hexaploide*) (Hosford, 1975), pea (*Pisum sativum*) (Tran et al., 2014), sunflower (*Helianthus annuus*) (Roustaee et al., 2000), grapevine (*Vitis vinifera*) (Khani, 2014), peach (*Prunus persica*) (Thomidis et al., 2011), kiwi (*Actinidia chinensis*) (Pan et al., 2018), pistachio (*Pistacia vera*) (Moral et al., 2018), Chinese cornel dogwood (*Cornus officinalis*) (Huang et al., 2018) and apple (*Malus* spp.).

During a disease survey in the 2021 growing season, we observed gray leaf blight symptoms on maize in Panjin, Liaoning, a northeastern province of China. The symptoms differed from those caused by *D. zeae-maydis*, the only *Didymella* species infectious on maize and the causal agent of yellow leaf blight. Culture characteristics and molecular phylogeny based on multi-locus DNA barcodes (ITS, *Actin* and *β-tubulin*) showed that the single spore culture isolate Pj-2 retrieved from the infected leaf tissues was morphologically and genetically identical with the *D. glomerata* isolate D/034 from soybean (*Glycine max*) (Irinyi et al., 2009). This was the first report of *D. glomerata* causing DLB on maize (Ma et al., 2022).

*D. glomerata* is likely a heterothallic fungal pathogen as it harbors the mating-type gene *scaffold1.t522* (*MAT1-2*), whose protein product contains the characteristic high mobility group box domain. The pathogen likely deploys a subcuticular intramural necrotrophic infection strategy, whereby the pathogen colonizes maize leaves, resulting in DLB. The infection cycle starts when fungal spores (conidia) land on the leaf surface and germinate to form a germ tube. However, unlike hemibiotrophic fungal pathogens, the germ tube does not differentiate into appressoria (specialized structures dedicated to mechanized penetration of the host surface) but instead directly perforates the host cuticle, presumably *via* an infection peg. The pathogen can also gain access to host tissues through natural openings, such as stomata. Following successful penetration, the invasive hyphae emanating from the infection pegs rapidly colonize the host parietal layers and eventually kill and macerate the host cells. Black lesions appear on the maize leaves five to seven days after infection. Single-celled ellipsoidal conidia are produced in glabrous pycnidia developing in these lesions and are dispersed by splashing raindrops and wind to infect healthy neighboring plants.

Thus far, studies have revealed that *D. glomerata* produces phytotoxins zinniol (Sugawara and Strobel, 1986) and 5-dihydroxymethylfuran (Cimmino et al., 2021), which contribute to the virulence of the pathogen on sunflowers and grapevine, respectively. However, molecular and genetic factors regulating the *D. glomerata* virulence on various hosts, let alone maize, are still lacking. Only seven species in the genus *Didymella* have been sequenced, and the genomes thereof are available in the public domain. Of the sequenced *Didymella* species, two are pathogenic to humans (*D. keratinophila* [the causal agent finger-hand lesions in humans; GenBank accession GCA_011058865.1] and *D. heteroderae* [GenBank accession GCA_011058895.1]), whereas five are pathogenic to plants (*D. pinodes* [the causal agent of Ascochyta blight of pea; GenBank GCA_004151525.1], *D. lethalis* [pea root rot pathogen; GenBank accession GCA_004335245.1], *D. arachidicola* [the causal agent of web blotch of peanut; GenBank accession GCA_016630955.1], *D. exigua* [the causal agent of Didymella wilt and necrosis of yellow starthistle; GenBank accession GCA_010094145.1] and *D. rabiei* [the causal agent of Ascochyta blight of chickpea; GenBank accession: GCA_004011695.1]). More recently, fragmented genome assemblies of two *D. glomerata* strains CBS 528.66 (accession GCA_022559905.1; host: *Chrysanthemum* spp.) and M27-16 (accession GCA_022225945.1; host: *Malus* spp.) were deposited to GenBank.

In this study, we sequenced, assembled and annotated the genome of *D*. *glomerata* strain Pj-2, which infects maize and causes DLB. Therefore, the high-quality gapless assembly reported in the study represents the first sequenced genome of the maize-infecting *D. glomerata* strain. Furthermore, we also identified three genetic sources of DLB resistance in maize germplasm, including an elite tropical inbred line CML496, for potential introgression of resistance alleles into elite cultivars lacking resistance through intraspecific hybridization.

## Results and discussion

### High-quality gapless genome assembly of maize-infecting *D. glomerata*

We sequenced the *D. glomerata* strain Pj-2 genome on the second-generation (Illumina NovaSeq 6000) and third-generation (PacBio Sequel II) sequencing platforms, which yielded a *de novo* reference genome assembly of 33.17 Mb with a 575-fold genomic coverage (**Table 1**). Thirty-two contiguous super-sequences (called supercontigs or scaffolds) were assembled from 2,054,785 clean SMRT reads (average read length = 8,300 bp; summed read length = 17,055,541,969 bp) originating from the sequencing of a long DNA fragment (insert size = 10 Kb) library on PacBio Sequel II. The scaffolds were confirmed and corrected by 250-bp 11,853,984 high-quality paired-end reads (summed length = 2,876,946,471 bp; average phred quality score on a sliding window of four nucleotides ≥30) generated from the sequencing of a short paired-end library (insert size = 500 bp) on NovaSeq 6000. As a result, a virtually gapless high-quality genome assembly was generated and consisted of 32 scaffolds with only a single gap of 100 bp in scaffold14 (coordinate: 515,915–516,014 bp). The analysis of *K*-mer frequency at the optimal length of 17 bp within 250-bp 11,853,984 paired-end reads revealed that the genome of Pj-2 is estimated to be 34.90 Mb in size. The genome size is comparable to the recently sequenced genome assemblies of the Chrysanthemum-infecting strain CBS 528.66 (33.98 Mb) and the apple-infecting strain M27-16 (35.52 Mb) of *D. glomerata*.

**Table 1:** The *Didymella glomerata* Pj-2 genome assembly features.

Genome features	Statistics
Assembly size	33,173,266 bp
Scaffolds	32
Scaffold N/L50	11/1,359,674 bp
Gap	100 bp
Protein-coding gene models	10,334
GC content	53.44%
tRNA	145
rRNA	43
ncRNA	31
Repeat elements	1.93%
Gene model coverage^a^	
Complete BUSCOs	6,480 (97.6%)
Fragmented BUSCOs	12 (0.2%)
Missing BUSCOs	149 (2.2%)

a The completeness of genome assembly was evaluated using a set of 6,641 pleosporales BUSCOs (benchmarking universal single-copy orthologs).

The scaffold N/L_50_ of the *D. glomerata* Pj-2 genome is 11/1,359,674 bp, which is far superior than the other two genome assemblies of *D. glomerata* strains CBS 528.66 (177 scaffolds; N/L_50_ = 7/635,919 bp) and M27-16 (946 scaffolds; N/L_50_ = 26/469,072 bp). In addition, the Pj-2 genome assembly is either comparable or better than that of other sequenced *Didymella* species: *D. keratinophila* 9M1 (577 scaffolds; N/L_50_ = 76/141,571 bp), *D. heteroderae* 28M1 (620 scaffolds; N/L_50_ = 38/283,873 bp), *D. pinodes* WTN-11-157 (1,593 scaffolds; N/L_50_ = 91/106,671 bp), *D. lethalis* GRM-16-623 (512 scaffolds; N/L50 = 50/196,696), *D. arachidicola* YY187 (25 scaffolds; N/L_50_ = 8/2,070,310 bp) (Li et al., 2021); *D. exigua* CBS 183.55 (176 scaffolds; N/L_50_ = 64/145,421 bp), *D. rabiei* ArMe14 (34 scaffolds; N/L_50_ = 9/1,812,190) (Shah et al., 2020) and *D. segeticola* GZSQ-4 (23 scaffolds; N/L_50_ = 6/2,254,797) (Ren et al., 2019).

Tandem telomeric repeats (CCCTAA)_n_ or (TTAGGG)_n_ were present in at least one end of the 23 scaffolds of the *D. glomerata* Pj-2 genome assembly. Of these 23 scaffolds, four (scaffold1 [1.90 Mb], scaffold14 [1.09 Mb], scaffold18 [1.05 Mb] and scaffold19 [1.03 Mb]) contain the telomeric repeats at both ends and hence represent full-length chromosomes. Transposable elements (TEs) comprise only 1.93% of the *D. glomerata* genome assembly (0.99% [328,869 bp] of retroelements, 0.90% [297,166 bp] of DNA transposons and 0.02% [7,437 bp] of unclassified TEs) and are scattered in the low GC content regions of the genome (**Table S1**). The GC content in the Pj-2 genome is 53.44%, which is similar to the CBS 528.66 (53.10%) and M27-16 (53.30%) genomes and other sequenced *Didymella* spp. A total of 6,480 (97.6%) out of 6,641 pleosporales (the order within Dothidiomycetes) single-copy genes were mapped on the *D. glomerata* Pj-2 genome, suggesting a near-complete genome assembly with regard to the genic content thereof. Overall, the assembled PacBio reads corrected by the Illumina reads produced a high-quality (32 scaffolds with 575-fold genome coverage), nearly gapless (single gap of 100 bp) genome assembly of the maize-infecting strain Pj-2 of *D. glomerata*.

### Gene families implicated in the virulence/pathogenicity of *D. glomerata* on maize

The *D. glomerata* Pj-2 genome is predicted to contain 10,334 protein-coding genes, whose number is markedly lower than that of the *Chrysanthemum*-infecting strain CBS 528.66 (11,200) and the *Malus*-infecting strain M27-16 (11,650). Similarly, the predicted gene content of Pj-2 encodes is markedly lower than that of other *Didymella* spp., e.g., *D. keratinophila* 9M1 (11,880), *D. heteroderae* 28M1 (11,640), *D. exigua* CBS 183.55 (12,356) and *D. rabiei* ArMe14 (11,257), except *D. pinodes* WTN-11-157 (10,505). The clustering of 90,823 proteins coded by the eight strains based on their sequence similarity (≥90% over 50 aligned aa) showed that the strains carry 1,027 core orthologous proteins. Pj-2 shares 2,734 more orthologous proteins with CBS 528.66 (7,660) than M27-16 (4,927), suggesting that the maize-infecting *D. glomerata* strains are phylogenetically closely related to the *Chrysanthemum*-infecting *D. glomerata* strain. Pj-2 shares 5,444 orthologous proteins with 9M1, 4,771 with 28M1, 3,847 with CBS 183.55 and 1,958 with ArMe14 (**Table S2**).

**Figure 1:**
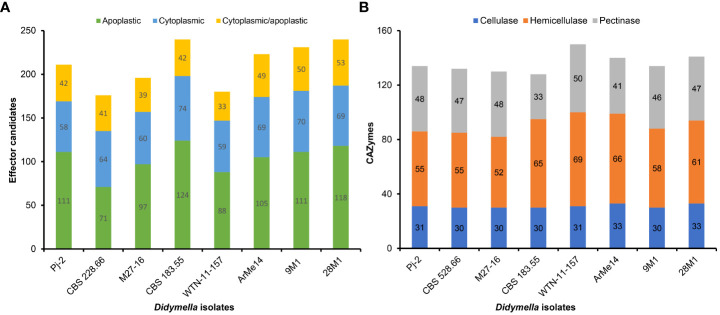
Distribution of effector candidates **(A)** and carbohydrate-active enzymes (CAZymes; **B**) in eight strains representing six *Didymella* spp. (*Didymella glomerata* Pj-2, CBS 528.66 and M27-16, *D. pinodes* WTN-11-157, *D. keratinophila* 9M11, *D. heteroderae* 28M1, *D. exigua* CBS 183.55 and *Ascochyta* [*Didymella*] rabiei ArMe14).

Only a subset of genes in the genomes encodes virulence/pathogenicity-related factors, including although not limited to effectors, carbohydrate-active enzymes (CAZymes) and secondary metabolism enzymes. Effectors are the pathogen-secreted small proteins and are an example of the extended phenotype as they function in hosts instead of pathogens, whose genomes carry the effector-coding genes. Pathogens deliver effectors into plants to subdue the innate immune system of plants, thereby enabling colonization and proliferation (Stergiopoulos and De Wit, 2009). Only 1.88% of the Pj-2 genes encode effector candidates (211), whose number is higher than that of the *Chrysanthemum*-infecting strain CBS 528.66 (176) and the *Malus*-infecting strain M27-16 (196) even though the latter two strains of *D. glomerata* have a larger gene content than the former (**Tables S3**-**S5**). Similarly, the pea-infecting *D. pinodes* WTN-11-157 genome carried a reduced number of effector candidate genes (180; **Table S6**). However, effector content in other four strains were slightly larger than that in Pj-2, e.g., 9M1 (231; **Table S7**), 28M1 (240; **Table S8**), CBS 183.55 (240; **Table S9**) and ArMe14 (223; **Table S10**). The majority of these effectors lack any known functional domain, e.g., 195 Pj-2 effectors show homology to hypothetical and uncharacterized proteins. Some of these effectors function in the host cell apoplast, which is replete with cysteine proteases, whereas some effectors are translocated into host cells wherein they subvert host cell immunity, viz., effector-triggered immunity (ETI). EffectorP (Sperschneider and Dodds, 2022) predicted that 111 encoded by the Pj-2 genome are likely translocated into the host cell apoplast. Seventy-four of the 111 apoplastic effector candidates possess ≥2% cysteine residues; cysteine residues in some of these effectors form disulfide bonds, which may provide stability to them in the hostile protease-rich milieu in the host cell apoplast (**Figure 1A**). The distribution of apoplastic effector candidates in the eight strains followed a similar pattern as effector candidates, i.e., Pj-2 carries a larger repertoire of apoplastic effector candidates than that in CBS 528.66 (71), M27-16 (97) and WTN-11-157 (88). A total of 58 effector candidates are likely transported into the host cell cytoplasm; notably, the number of these cytoplasmic effector candidates is lower than that in CBS 528.66 (64), M27-16 (60) and WTN-11-157 (59) (**Figure 1A**). Effectors in some plant pathogens, such as *Leptosphaeria maculans* (the causal agent of blackleg disease of oilseed crop), *Fusarium* spp., *Magnaporthe oryzae* (the causal agent of rice blast disease) and *Ustilago maydis* (the causal agent of corn smut), are localized in chromosomal regions enriched with transposable elements, including telomeres, or clustered in AT-rich blocks of the genomes (Kämper et al., 2006; Farman, 2007; Ma et al., 2010; Rouxel et al., 2011). Such a genomic environment drives rapid diversification of genes, including those coding for effectors, thereby enabling pathogens to evade the innate immune system of host cultivars. However, the effector candidates in Pj-2 are randomly distributed across the genome, with no evidence for enrichment of particular scaffolds, including full-length chromosomes (scaffold1, scaffold14, scaffold18 and scaffold19), or clustering in the proximity of genomic regions replete with repetitive elements, e.g., telomeres. The relatively larger effector repertoire of Pj-2, especially cytoplasmic effectors, may be an adaptation of *D. glomerata* to colonize new host maize under field conditions, which we reported recently (Ma et al., 2022).

Pj-2 is equipped with 134 carbohydrate-active enzymes (CAZymes; **Figure 1B** and **Table S11**) that likely degrade the host cell wall, composed mainly of cellulose, hemicellulose and pectin, to colonize the host tissues and drive nutrition from the macerated tissues (van den Brink and de Vries, 2011). The other two *D. glomerata* strains possess a slightly lower number of CAZymes, i.e., CBS 528.66 (132; **Figure 1B** and **Table S12**) and M27-16 (130; **Figure 1B** and **Table S13**). The number of CAZymes in other *Didymella* spp. (**Figure 1B** and **Tables S1**-**S17**) except CBS 183.55 (128; **Figure 1B** and **Table S18**) is higher than that in Pj-2. Notably, the number of hemicellulases (55) is higher than cellulases (31) and pectinases (48) in Pj-2, which is presumably associated with a higher amount of hemicellulose in the maize cell wall.

In fungal pathogens, secondary metabolism genes are generally localized in biosynthetic gene clusters (BGCs), and each BGC contains a backbone gene and a few decorating genes, and encodes a specific secondary metabolite (Collemare et al., 2008). Secondary metabolites are indispensable for fungal virulence on hosts (Soanes et al., 2012). The Pj-2 genome contains 12 BGCs, similar to CBS 528.66 (12) but lower than M27-16 (16) (**Tables 2**, **S19**-**S26**). Likewise, the number of backbone genes encoding polyketide synthase (PKS) and nonribosomal peptide synthase (NRPS) is slightly higher in M27-16 (6 PKS and 7 NRPS-like) than Pj-2 (4 PKS and 4 NRPS-like) and CBS 528.66 (5 PKS and 4 NRPS-like) (**Tables 2** **,** **S19**-**S26**). Therefore, the *Malus*-infecting *D. glomerata* strain M27-16 may have more capacity to produce secondary metabolites than that of the maize- and *Chrysanthemum*-infecting *D. glomerata* strains.

**Table 2:** Biosynthetic gene clusters (BGCs) and secondary metabolite backbone-forming enzymes within BCGs in *Didymella* spp.

	Pj-2^3^	CBS 528.66^3^	M27-16^3^	CBS 183.55^4^	WTN-11-157^5^	ArMe14^6^	9M1^7^	28M1^8^
BGCs^1^	12	12	16	12	15	22	10	13
Genes in BGCs								
PKS	4	5	6	8	8	12	4	7
PKS-like	1	1	1	2	1	1	2	1
NRPS	2	2	2	3	2	2	2	2
NRPS-like	4	4	7	3	5	6	4	4
PKS-NRPS	0	0	0	0	0	1	0	0
DMAT	1	1	1	1	1	1	0	1
Total^2^	12	13	17	17	17	23	12	15

^1^BGCs, Biosynthetic gene clusters.

^2^Total number of secondary metabolite backbone-forming enzymes.

^3^Didymella glomerata isolates Pj-2 (sequenced in this study), CBS 528.66 and M27-16.

^4^Didymella exigua isolate CBS 183.55.

^5^Didymella pinnodes isolate WTN-11-157.

^6^Ascochyta (Didymella) rabiei isolate ArMe14.

^7^Didymella keratinophila isolate 9M1.

^8^Didymella heteroderae isolate 28M1.

### Phylogenomics reveals intra- and inter-specific genetic diversity among *Didymella* spp.

The *D. glomerata* strain Pj-2 genome (33.17 Mb) is similar in size to the Chrysanthemum-infecting strain CBS 528.66 (33.98 Mb), apple-infecting strain M27-16 (35.52 Mb), as well as other *Didymella* spp., e.g., *D. pinodes* WTN-11-157 (33.77 Mb), and *D. exigua* CBS 183.55 (34.39 Mb). However, the other four sequenced *Didymella* spp. have slightly larger genomes, e.g., *D. rabiei* ArMe14 (40.92 Mb), *D. keratinophila* 9M1 (36.22 Mb) and *D. heteroderae* 28M1 (37.07 Mb).

**Figure 2:**
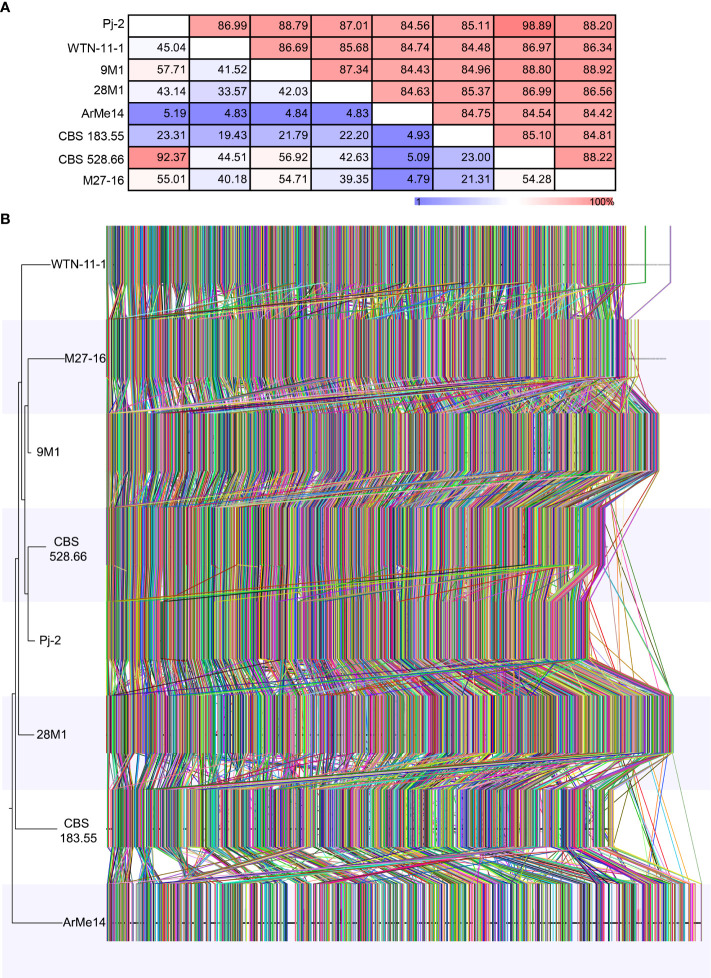
Phylogenomics of *Didymella* spp. The whole genomes of eight *Didymella* spp. strains are aligned using CLC Genomic Workbench with 50 bp seeds: *D. glomerata* Pj-2 (33.17 Mb; 32 scaffolds), CBS 528.66 (33.98 Mb; 177 scaffolds) and M27-16 (35.52 Mb; 946 scaffolds), *D. keratinophila* 9M1 (36.22 Mb; 577 scaffolds), *D. heteroderae* 28M1 (37.07 Mb; 620 scaffolds), *D. pinodes* WTN-11-157 (33.77 Mb; 1,593 scaffolds), *D. exigua* CBS 183.55 (34.39 Mb; 176 scaffolds) and *D. rabiei* ArMe14 (40.92 Mb; 34 scaffolds). The seeds were extended using a HOXD scoring matrix until the local alignment score dropped. Pairwise distance matrices between the aligned genomes are calculated from the alignment blocks of size ≥100 bp and are expressed as alignment percentage (AP; upper) and average nucleotide identity (ANI; lower) **(A)**. Genome-wide synteny analysis among the eight strains **(B)**. Each colored link represents a syntenic block (≥1,000 bp) that is shared among genomes. A neighbor-joining tree is also shown to the left of the whole-genome alignment.

To gauge genetic diversity among *Didymella* spp., we performed genome similarity and synteny analyses among eight strains, representing six species. An eight-strain whole-genome alignment was contrived using the following parameters: 50-bp seeds and extension thereof with HOXD scoring matrix (Chiaromonte et al., 2002) and ≥100-bp alignment block. Pairwise distance matrices between the genomes were calculated from the alignment blocks of size ≥100 bp and were expressed as alignment percentage (AP) and average nucleotide identity (ANI) (**Figure 2B**). The *D. glomerata* strain Pj-2 shares 92.37% (i.e., AP) of its genome with CBS 528.66, followed by 57.71% with 9M1, 55.01% with M27-16, 45.04% with WTN-11-157, 43.14% with 28M1, 23.31% with CBS 183.55 and 5.19% with ArMe14. The shared genomic regions showed ≥85% ANI; Pj-2 displayed 98.89% nucleotide similarity with CBS 528.66, followed by 88.79% with 9M1, 88.20% with M27-16, 87.01% with 28M1, 86.99% with WTN-11-1, 85.11% with CBS 183.55 and 84.56% with ArMe14. A neighbor-joining tree constructed from the pairwise distance matrices revealed that a clade comprising the three *D. glomerata* strains Pj-2, CBS 528.66 and M27-16, and *D. keratinophila* 9M1. These strains share 55.01% to 92.37% genomes (AP), with ANI values ranging from 88.20% to 98.89%. Within this clade, Pj-2 and CBS 528.66 formed a subclade, which is in agreement with high AP (92.37%) and ANI (98.89%) between their genomes (**Figure 2B**). Synteny analysis showed that the two *D. glomerata* strains possess a similar genomic architecture. However, a few structural variations (chromosomal rearrangements) exist between their genomes (**Figure 2B**). To further investigate these genomic rearrangements, whole-genome collinearity analysis was performed between the Pj-2 and the genomes of seven strains (CBS 528.66, M27-16, WTN-11-15, CBS 183.55, ArMe14, 9M1 and 28M1) (**Figure 2**). The Pj-2 genome is collinear with the CBS 528.66 genome, whereas it shows fragmented collinearity with the other genomes (due to low and extensive structural variations), including M27-16, which precluded further investigation (**Figures 2**, **S1**).

**Figure 3:**
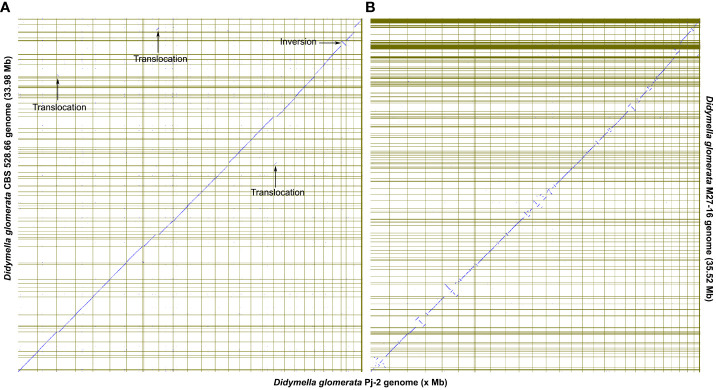
The dot plots show the collinearity of the *D. glomerata* Pj-2 genome (X-axis) with that of CBS 528.66 (Y-axis; **A**) and M27-16 (Y-axis; **B**). Four translocations are conspicuously present between Pj-2 and CBS 528.66.

Although the dot plot shows a high degree of collinearity between the two *D. glomerata* strains Pj-2 and CBS 528.6; however, there exist four major reciprocal translocations, viz. Pj-2 scaffold14/CBS 528.66 scaffold12 and Pj-2 scaffold18 (274 kb), Pj-2 scaffold22/CBS 528.66 scaffold6 and Pj-2 scaffold3 (166 kb), Pj-2 scaffold26/CBS 528.66 scaffold17 and Pj-2 scaffold13 (131 kb translocation), and Pj-2 scaffold26/CBS 528.66 scaffold17 and Pj-2 scaffold8 (217 kb translocation) (**Figure 3A**).

Pj-2 scaffold14 (size = 1094.13 kb; full-length chromosome) and CBS 528.66 scaffold12 (size = 763.58 kb) are collinear with a high degree of similarity in the genomic region located between 282 kb and 764 kb, whereas a 274-kb genomic region of CBS 528.66 scaffold12 (8 to 282 kb) shows alignment with a high degree of homology with a similar size genomic region located at the 5’-end of Pj-2 scaffold18 (size = 1051.93 kb; full-length chromosome). This is called the reciprocal translocation between Pj-2 scaffold14/CBS 528.66 scaffold12 and Pj-2 scaffold18. The 274 kb translocation region contains 92 genes in Pj-2 and 100 genes in CBS 528.66, 78 of which are homologous. This translocation encodes three apoplastic effector candidates with unknown functions. The Pj-2 effector candidates scaffold18.g41 (141 aa) and scaffold18.g46 (238 aa) are 100% identical to CBS 528.66 scaffold12.g4610 and scaffold12.g4605, respectively. The third effector candidate scaffold18.g5 (269 aa) is aligned with 100% identity from 6 aa to 265 aa of the 526 aa CBS 528.66 protein scaffold12.g4649 (526 aa); likewise, the third CBS 528.66 effector scaffold12.g4639 (186 aa) is matched with 97% identity from 1 aa to 184 aa of the 675 aa Pj-2 protein scaffold18.g16. Therefore, these proteins represent the truncated version of larger proteins and thus are unlikely effector candidates. In addition to effector candidates, the translocation codes for three CAZymes. The Pj-2 cellulase scaffold18.g13 (394 aa) and the CBS 528.66 protein scaffold12.g4641 (439 aa) are homologous proteins, showing 98% identity over the first 327 aligned aa, whereas the C-termini thereof are highly variable and former lacks 45 aa in this region. The Pj-2 hemicellulase scaffold18.g6 (837 aa) is homologous to the CBS 528.66 hemicellulase scaffold12.g4648 (837 aa), exhibiting 99% identity over the entire length of proteins. The Pj-2 pectinase scaffold18.g16 (675 aa) and the CBS 528.66 scaffold12.g4639 (186 aa) are homologous proteins with 99% identity over the first 184 aligned aa; however, scaffold12.g4639 was predicted as an effector candidate due to its length ≤300. Therefore, scaffold12.g4639 is a truncated pectinase rather than an effector candidate. The translocation does not carry any secondary metabolite backbone enzyme.

Pj-2 scaffold22 (size = 915.48 kb) and CBS 528.66 scaffold6 (size = 1123.25 kb) are collinear with a high degree of similarity in the genomic region located between the 7–911 kb (Pj-2 scaffold22) and 174–911 Kb (CBS 528.66 scaffold6); however, a 166-kb genomic region of CBS 528.66 scaffold12 (8–174 kb), which Pj-2 scaffold22 lacks, aligns with a high degree of homology with a similar size to the genomic region (25–191 kb) located at the 5’-end of Pj-2 scaffold3 (size = 1813.70 kb). This is the reciprocal translocation between Pj-2 scaffold22/CBS 528.66 scaffold6 and Pj-2 scaffold3. The 166 kb translocation contains 54 genes in Pj-2 and 60 genes in CBS 528.66, 53 of which are homologous. This translocation encodes two apoplastic and/or cytoplasmic effector candidates with unknown functional domains and one CAZyme hemicellulase; however, it does not code for any secondary metabolite backbone enzyme. The Pj-2 effector candidates scaffold3.g27 (226 aa; apoplastic/cytoplasmic localization) and scaffold3.g51 (300 aa; apoplastic localization) are 100% identical to the CBS 528.66 effector candidates sacffold6.g2757 and scaffold6.g2777, respectively. The Pj-2 hemicellulase scaffold3.g49 (338 aa) is homologous to the CBS 528.66 hemicellulase scaffold6.g2776 (1120 aa), displaying 99% over the 334-aligned aa, whereas the former lacks the remaining aa. Therefore, scaffold3.g49 is a truncated protein.

Pj-2 scaffold26 (size = 703.34 kb) and CBS 528.66 scaffold17 (size = 703.44 kb) are collinear with a high degree of similarity in the genomic region located between 1–277 kb (Pj-2 scaffold22) and 434–703 Kb (CBS 528.66 scaffold6). The remaining genomic region of Pj-2 scaffold26, spanning from 277 kb to 703 kb, is collinear with CBS 528.66 scaffold29 (size = 481.49 kb). However, a majority of the 430-kb genomic region located at the 5’-end of CBS 528.66 scaffold17 (15–430 kb), which Pj-2 scaffold26 lacks, matches with a high degree of homology with two genomic regions located at distinct Pj-2 scaffolds, i.e., scaffold13 (1,133–1,264 kb) and scaffold8 (1,222–1,439 kb). Therefore, there are two reciprocal translocations: Pj-2 scaffold26/CBS 528.66 scaffold17 and Pj-2 scaffold13 (131 kb translocation) and Pj-2 scaffold26/CBS 528.66 scaffold17 and Pj-2 scaffold8 (217–250 kb translocation). The 131-kb translocation contains 46 genes in Pj-2 and 55 genes in CBS 528.66, 45 of which are homologous genes. The translocation codes for one apoplastic and/or cytoplasmic effector candidate with an unknown functional domain; however, it does not encode

CAZymes and secondary metabolite backbone enzymes. The Pj-2 effector candidate scaffold13.g401(239 aa) is homologous to CBS 528.66 Scaffold17.g5676, showing 100% identity. The 217–250 kb translocation contains 77 genes in Pj-2 and 89 genes in CBS 528.66 (146-396 kb; scaffold17 of CBS 528.66), 77 of which are homologous genes. The translocation codes for two apoplastic and/or cytoplasmic effector candidates with an unknown function; however, it does not encode CAZymes and secondary metabolite backbone enzymes. The 136 aa effector candidates scaffold8.g410 (Pj-2) and scaffold17.g5766 (CBS 528.66) are homologous proteins, exhibiting 95% identity over their entire length; seven aa differ between the two effector candidates. Both are predicted to be localized in the apoplast and/or cytoplasm. However, the second effector candidate (294 aa; scaffold8.g423 in Pj-2 and scaffold17.g5781 in CBS 528.66) does not show any polymorphism between the two strains and is likely localized in the cytoplasm.

A spurious inversion is evident in the collinearity plot between Pj-2 and CBS 528.66. Pj-2 scaffold27 is flipped with respect to the 3’-end of CBS 528.66 scaffold4; the 3’-end of CBS 528.66 scaffold4 is aligned with Pj-2 scaffold 24. Therefore, we conclude that Pj-2 scaffold24 and scaffold27 are part of the same contiguous sequence.

The structural variations in the genomes are the major drivers of genetic diversity within and between species of the same genus, which enable the species or strains within the same species to colonize distinct ecological niches. For example, the maize- and Chrysanthemum-infecting strains of *D. glomerata* are genetically similar, sharing 92.37% genome with 98.89% sequence similarity. However, the *Malus*-infecting strain of *D. glomerata* M27-16 is highly diverse from the other two strains, Pj-2 (sharing only 55.01% genome with 88.20% sequence similarity) and CBS 528.66 (sharing 54.28% genome with 88.22% sequence similarity) (**Figures 2A**, **3B**). The four translocations between Pj-2 and CBS 528.66 may be responsible for the two strains colonizing different hosts, *Z. mays* and *Chrysanthemum* spp. However, it is unlikely that CAZymes and secondary metabolism genes located the translocations. We found only one effector candidate showing polymorphism between the two strains, Pj-2 (scaffold8.g410) and CBS 528 (scaffold17.g5766). It would be interesting to investigate whether the targeted deletion of these two effectors in their respective strains contributes to virulence on their hosts.

### Germplasm screening to identify the genetic sources of DLB resistance in maize

**Figure 4:**
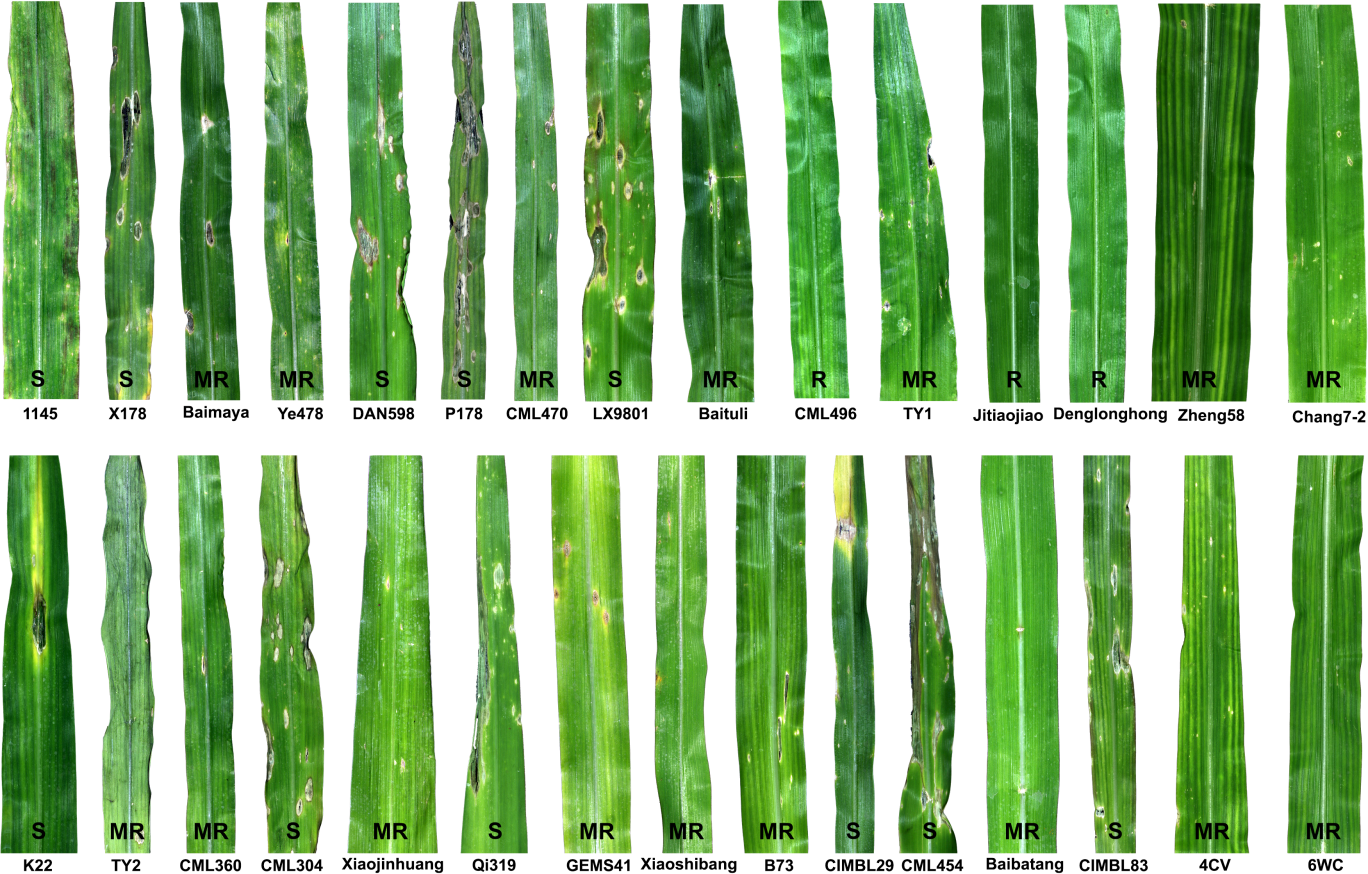
Maize germplasm screening to identify the genetic sources of resistance to *Didymella* leaf blight caused by *Didymella glomerata*. Fourth leaves of thirty lines (cultivars, inbred lines and landraces) at the V3 stage were spray-inoculated with conidial suspension (1 x 10^7^ conidia/ml in 0.025%) until runoff. Three of the lines expressed complete resistance (R; no lesion) to *D. glomerata*, followed by respectively sixteen and twelve lines exhibiting moderate resistance (MR; few confined scattered lesions) and susceptible (S; coalesced lesions) reactions. An augmented randomized complete block design was used to evaluate the maize germplasm for DLB resistance. The leaves were photographed five days post-inoculation.

Deployment of resistant cultivars in the field offers an environmentally friendly way to control crop diseases and thus reduces the overreliance on pesticides, thereby increasing farm crop cash receipts. However, developing a resistant cultivar requires an intraspecific hybridization through the wide/top cross between a susceptible cultivar (elite cultivar) and a donor parent (inbred line, landrace or wild relative) that carries alleles conferring genetic resistance to the disease of interest. Identifying such genetic sources of disease resistance is the first key step in cultivar development. With the above objective and given that DLB is a new disease of maize, we conducted germplasm screening to identify the genetic source of DLB resistance in maize. Thirty maize lines representing inbred lines, cultivars and landraces were screened for reactions to the *D. glomerata* isolate Pj-2 in a growth chamber through spray-inoculation following an augmented randomized block design (**Table S27**). Three of these lines expressed complete resistance (immune; no lesion) to *D. glomerata*, followed by respectively sixteen and eleven lines exhibiting moderate resistance (few confined scattered lesions) and susceptible (coalesced lesions) reactions (**Figure 4** and **Table S27**). The resistant lines were CML496, Jitiaojiao and Denglonghong; CML496 is an elite tropical inbred line, developed by CIMMYT, whereas the other two are local cultivars. CML496 is highly resistant to *Puccinia polysora*, the causal agent of southern corn rust (SCR) and carries a major QTL *RppCML496* for resistance to SCR (Lv et al., 2021). Among the moderately resistant lines were elite inbred lines, such as Ye478 (lesion area/2500 mm^2^: 12.7 ± 19.2 mm^2^), Zheng58 (16.7 mm^2^), Chang7-2 (39.7 mm^2^), CML470 (51.0 mm^2^), PH4CV (32.6 mm^2^), PH6WC (6.5 mm^2^) and B73 (66.6 mm^2^) (**Figure S2** and **Table S27**). Ye478, Zheng58 and Chang7-2 are elite lines, heavily used as parental lines in hybrid breeding programs in China because of their high general combining ability and superior agronomic performance (Zheng et al., 2009). All three lines display moderate resistance to *Bipolaris maydis* (the causal agent of southern corn leaf blight) and *Puccinia sorghi* (common rust pathogen), whereas they are susceptible to *Curvularia lunata* (the causal agent of Curvularia leaf spot) and *P. polysora*. In addition, Zheng58 and Chang7-2 are moderately resistant to *Exserohilum turcicum* (northern corn leaf blight pathogen), and the latter line also manifests a similar resistance response to *Cercospora zeae-maydis* (gray leaf spot pathogen) (Wang et al., 2014). CML470, a CIMMYT inbred line, carries a dominant gene *RppC*, which controls resistance to *P. polysora (* Sun et al., 2020). PH4CV and PH6WC are the elite inbred lines developed by Pioneer HiBred International; however, their reactions to fungal pathogens are largely unexplored. B73 is susceptible to *B. maydis*, *C. lunata*, *C. zeae-maydis* and *P. polysora*, whereas the line shows moderate resistance to *E. turcicum* and complete resistance to *P. sorghi*.

**Figure 5:**
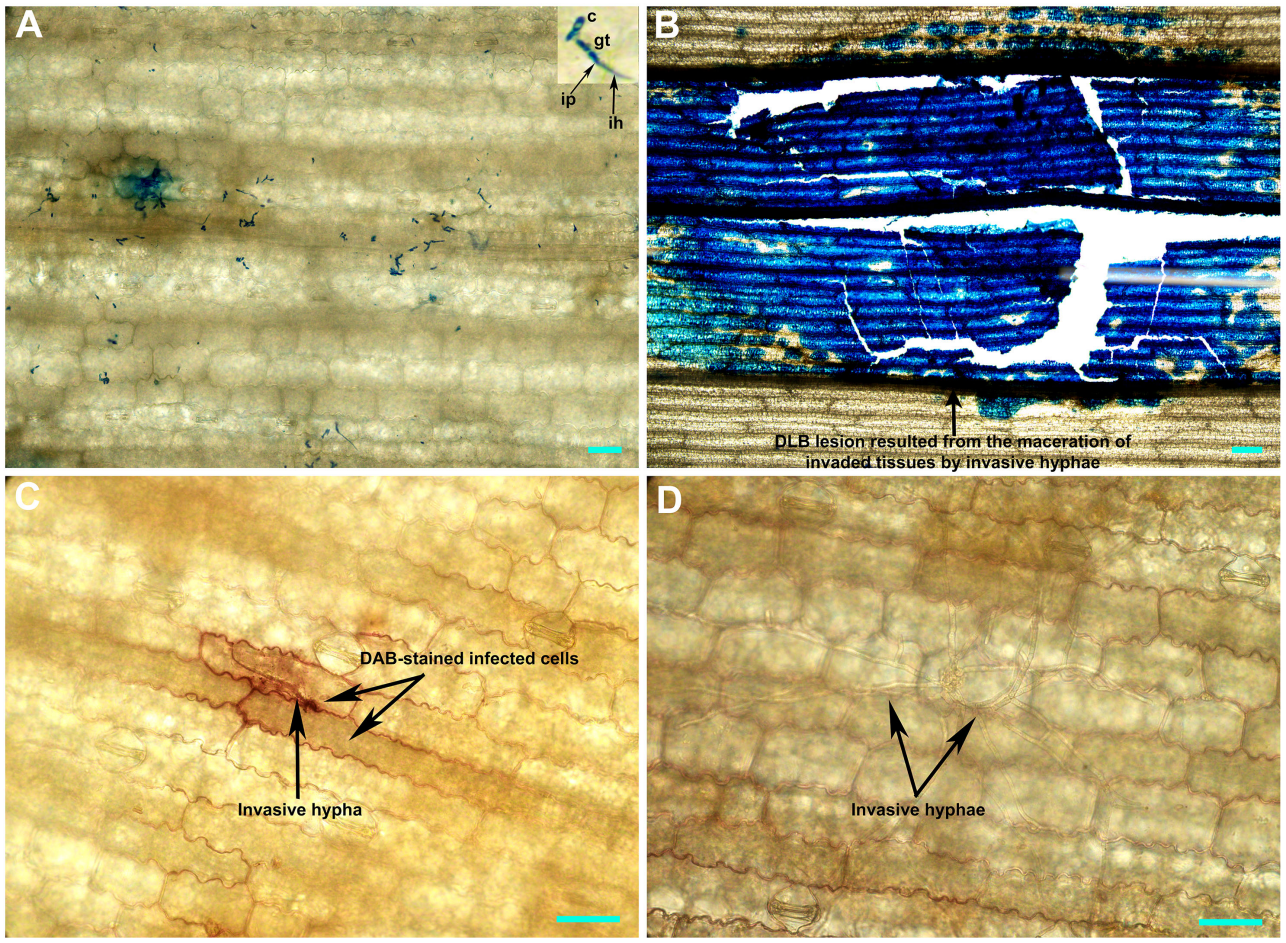
Histochemical staining of the *Didymella glomerata* strain Pj-2-infected tissues of susceptible (P178) and resistant (CML496) inbred lines of maize. The top image panel shows the acidic aniline blue-stained CML496 **(A)** and P718 **(B)** leaf tissues, and the bottom image panel illustrates the DAB-stained CML496 **(C)** and P178 **(D)** leaf tissues five days post-inoculation. The *D. glomerata* conidia germinate to form germ tubes, which directly breach the host cell cuticle, presumably *via* infection pegs. This invasion is restricted to the first infected epidermal cells **(A)** and leads to the accumulation of H_2_O_2_ (a reactive oxygen species; c) in CML496, whereas the invasion in P178 leads to the maceration of infected tissues **(B)** by invasive hyphae **(D)**. c, conidia; gt, germ tube; ip, infection peg; ih, invasive hypha; Bars = 20 µm.

Furthermore, we selected the most susceptible inbred line (P178) and the most resistant inbred line (CML496) for histochemical analysis. P178 is an inbred line, which is resistant to *P. polysora* SCR (six QTL for SCR resistance) (Tian et al., 2018) and *Exserohilum turcicum (* Wang et al., 2019). However, P178 exhibited extensive leaf blight following the inoculation with the *D. glomerata* strain Pj-2; over 50% of the inoculated leaf area showed blight (lesion area/2500 mm^2^: 1278.6 ± 38.5 mm^2^) while the CML496 leaves remained healthy (**Figures 4** **,** **S2** and **Table S27**). DAB-staining of the *D. glomerata*-infected leaf tissues revealed the accumulation of H_2_O_2_, a reactive oxygen species, in the apoplastic space of the infected issues of CML496, not in P718 (**Figure 5**). The rapid and transient burst of ROS (called oxidative burst) at the infection site is the hallmark of the pathogen-associated molecular pattern (PAMP)-triggered immunity (PTI) and ETI, and is ensued upon successful recognition of pathogens by host cells (Torres, 2010). The CML496 infected cells likely successfully perceived *D. glomerata*, which in turn activated defense responses, such as oxidative burst that arrested fungal growth and proliferation (**Figures 5A, C**). The pathogen apparently requires successful penetration of the host epidermal cell cuticle, which may trigger its perception in the CML496 epidermal cells, leading to DLB resistance (**Figure 5C**). However, *D. glomerata* likely dodges surveillance (PTI and ETI) in the P178 epidermal cells, facilitating fungal growth and proliferation (**Figures 5B, D**). Altogether, the DLB of maize is likely a gene-for-gene disease in which the resistant maize genotypes (e.g., CML496, Jitiaojiao and Denglonghong) and *C. graminicola* recognize each other by corresponding pairs of the nucleotide-binding leucine-rich repeat receptors and effectors.

## Concluding remarks

DLB is a new disease of maize, which we recently identified in Panjin, Liaoning, a northeastern province of China (Ma et al., 2022). In this study, we presented a nearly gapless genome assembly of the *D. glomerata* strain Pj-2, which comprises 32 scaffolds, including four full-length scaffolds (representing chromosomes) carrying telomeres at both ends. Phylogenomic analysis shows that the maize- and *Chrysanthemum*-infecting strains of *D. glomerata* are genetically similar and share 93.37% genome with 98.89% sequence similarity. However, they carry four major reciprocal translocation regions in their genomes, which may allow their adaptation to different hosts. Furthermore, germplasm screening against DLB identified three maize inbred lines (e.g., CML496, Jitiaojiao and Denglonghong) expressing complete resistance to the *D. glomerata* strain Pj-2. Therefore, these lines can be used as a donor parent to transfer genetic resistance into other elite cultivars lacking DLB resistance through intraspecific hybridization introgression. Further research, however, is required to identify genes in CML496 conferring resistance to DLB. In addition, field research should be undertaken to assess the economic impact of this new maize disease, both in terms of yield loss and down-gradation of grain quality.

## Methods

### Fungal and plant material

The *D. glomerata* strain Pj-2 was routinely maintained on PDA plates under a 12 h photoperiod at 25°C. Maize germplasm involving 30 lines representing cultivars, inbred lines and landraces (**Table S27**) was evaluated for reactions to Pj-2.

### Genome sequencing, size, assembly and completeness

Conidia (ca. 10^7^) were collected by swamping the five-day-old six-cm PDA culture plates of Pj-2 with five ml of distilled deionized H_2_O. One ml of conidial suspension was incubated in a one-liter flask containing 200 ml of complete medium for four days in an orbital shaker set at 25°C and 120 rpm. Mycelia balls were collected by filtering the liquid culture through a one-layered Miracloth. The ENZA Fungal DNA kit (Omega Bio-Tek, Norcross, USA) was used to isolate genomic DNA (gDNA) from the mycelia.

Covaris E220 (Covaris, Brighton, UK) was used to fragment one µg of high-quality gDNA, and end-repaired fragments were used to construct a short insert (500 bp) paired-end library using TruSeq DNA LT Kit (Illumina, San Diego, USA). The library was assessed for the insert size and quality using BioAnalyzer 2100 using Agilent 1000 DNA Chip (Agilent Technologies, Palo Alto, USA) and sequenced on NovaSeq 6000 (Illumina, San Diego, USA), generating 2 x 250 bp reads. AdapterRemoval v2 (Schubert et al., 2016) was used to clip adapters and low-quality bases from the paired-end reads. SOAPec v2.0 (Luo et al., 2012) was used to correct sequencing errors based on the *K*-mer (17) frequency in the paired-end reads. Finally, Jellyfish v2.0 (Marçais and Kingsford, 2011) was used to count *K*-mer (17) within the high-quality paired-end reads to estimate the *D. glomerata* Pj-2 genome size.

g-TUBE (Covaris, Brighton, UK) was used to shear five µg of high-quality gDNA, and BluePippin (Sage Science, Beverly, USA) was utilized to size-select ~10 kb gDNA fragments. Finally, the end-repaired large fragments were used to construct a long insert (10 kb) library using SMRTbell Express Template Prep Kit 2.0 (Pacific Biosciences, Menlo Park, USA). The resulting library was assessed for insert size and quality on BioAnalyzer 2100 and sequenced on PacBio Sequel II (Pacific Biosciences, Menlo Park, USA) using Continuous Long Read mode. The polymerase reads generated on PacBio Sequel II were checked for adapter contamination, quality and size using SMRT analysis v2.3.0; the subreads (quality >0.8 and size >1,000 bp) were extracted from the adapter-free polymerase reads. Unicycler (Wick et al., 2017) was employed to contrive a hybrid genome assembly using high-quality 250-bp paired-end reads (NovaSeq 6000) and 1,000–10,000 bp subreads (PacBio Sequel II).

### Phylogenomics

The whole genomes of eight strains of six *Didymella* spp. (*D. glomerata* Pj-2, CBS 528.66 and M27-16, *D. keratinophila* 9M1, *D. heteroderae* 28M1, *D. pinodes* WTN-11-157, *D. exigua* CBS 183.55 and *D. rabiei* ArMe14) were aligned using the Whole Genome Alignment tool plugged in CLC Genomics Workbench (Qiagen, Aarhus, Denmark) with the following parameters: 50-bp seed and minimum alignment block size ≥100 bp. AP and ANI were calculated from the whole-genome alignment using pairwise distance matrices.

### Genome annotation

The following genomes were masked for repetitive DNA elements using RepeatMasker v4.0.6 (Smit et al., 1996) and RepeatModeler v2.1 (Smit and Hubley, 2015): the three *D. glomerata* strains (Pj-2, CBS 528.66 and M27-16), *D. exigua* CBS 183.55, *D. pinodes* WTN-11-157, *D. rabiei* ArMe14, *D. keratinophila* 9M1 and *D. heteroderae* 28M1. The masked genome of Pj-2 was assessed for its completeness using BUSCO v5.1.3 (Simão et al., 2015) with a set of 6,641 benchmarking universal single-copy orthologs (BUSCOs) in Pleosporales.

Augustus v3.03 (Stanke and Morgenstern, 2005), glimmerHMM v3.0.1 (Majoros et al., 2004) and GeneMark-ES v4.35 (Ter-Hovhannisyan et al., 2008) were used to predict gene models *ab initio* in the masked genomes. Protein models from the closely related *Didymella* species (*D. keratinophila*, *D. heteroderae*, *D. exigua* and *D. rabiei*) were mapped onto the *D. glomerata* genome using Exonerate v2.2.0 (http://www.ebi.ac.uk). The *ab initio* predictions and the Exonerate alignments were combined using EVidenceModeler v.r2012-06-25 (Haas et al., 2008) to obtain final gene models. CAZymes and secondary metabolism genes were predicted as described previously (Bhadauria et al., 2019). Genes that encode protein equal to or less than 300 aa in length, carrying N-terminal signal peptide (predicted using SignalP v5.0), lacking transmembrane domain (TMHMM v2.0) and GPI anchor addition site (PredGPI) were considered as effector candidate genes. In addition, EffectorP v3.0 was employed to discern their potential localization in the host cells, i.e., apoplast, cytoplasm and apoplast/cytoplasm. We also screened the effector candidates for possible N- and O-glycosylation sites using NetNGlyc v1.0 and NetOGlyc v4.0, respectively, as such sites allow their tethering to plasma membrane.

### Germplasm screening and statistical analysis

Thirty maize lines were individually grown in pots (6 x 10 cm) containing peat moss (soilless medium) and perlite in a 2:1 ratio in a growth chamber following an augmented randomized block design. Plants were spray-inoculated at the V3 growth stage with the Pj-2 conidia (1 x 10^7^ conidia/ml) following the method described previously (Vargas et al., 2012). The fourth leaves of the inoculated plants were photographed six days post-inoculation (dpi), and the lesion area (mm^2^) was measured using ImageJ (https://imagej.net/). A linear mixed-effects model using the R lm() function was used to calculate least-squares (LS) means of lesion areas on the maize lines (a fixed effect) while considering blocks (biological replications) as a random effect.

### Histochemical analysis of the Pj-2 infected maize tissues

Acidic aniline blue and 3, 3-diaminobenzidine (DAB) staining of Pj-2 infected leaf tissues of the inbred lines P178 and CML496 were performed by methods described by Bhadauria et al. (2010) and Thordal-Christensen et al. (1997), respectively.

## Data availability statement

The *D. glomerata* Pj-2 (BioSample: SAMN23554018) genome (32 scaffolds) has been deposited to GenBank/ENA/DDBJ under the accession number JAJOHN000000000 (BioProject: PRJNA785361).

## Author contributions

VB conceived and performed research. Y-LP, VB, JD, WZ, JY and WM analyzed data. JD provided maize germplasm and VB, Y-LP, JD, WZ and JY wrote the paper. All authors contributed to the article and approved the submitted version.

## Acknowledgments

The research was supported by grants from the National Natural Science Foundation of China (Grant No. 32172363) and the Chinese Universities Scientific Fund (Grant No. 10092004). Technical support for the incumbent study was provided by Hongjie Yuan at the Communication University of China, Beijing, China.

## Conflict of interest

The authors declare that the research was conducted in the absence of any commercial or financial relationships that could be construed as a potential conflict of interest.

## Publisher’s note

All claims expressed in this article are solely those of the authors and do not necessarily represent those of their affiliated organizations, or those of the publisher, the editors and the reviewers. Any product that may be evaluated in this article, or claim that may be made by its manufacturer, is not guaranteed or endorsed by the publisher.

## Supplementary material

Supplementary Figure 1 Genome collinearity of D. glomerata Pj-2 with Didymella keratinophila 9M1, Didymella heteroderae, Didymella pinnodes WTN-11-157, Didymella exigua CBS 183.55 and the Didymella (Ascochyta) rabiei ArMe14.

Supplementary Figure 2 Reactions of the *Didymella glomerata* strain Pj-2 on 30 maize lines representing cultivars, inbred lines and landraces five days post-inoculation. Lesion areas were measured using ImageJ (https://imagej.net/).
