# Supplementary figures and images for: Gapless reference genome assembly of Didymella glomerata, a new fungal pathogen of maize causing Didymella leaf blight
Source: Front Plant Sci. 2022 Oct 26;13:1022819. doi: 10.3389/fpls.2022.1022819 (PMC9643772; doi:10.3389/fpls.2022.1022819)

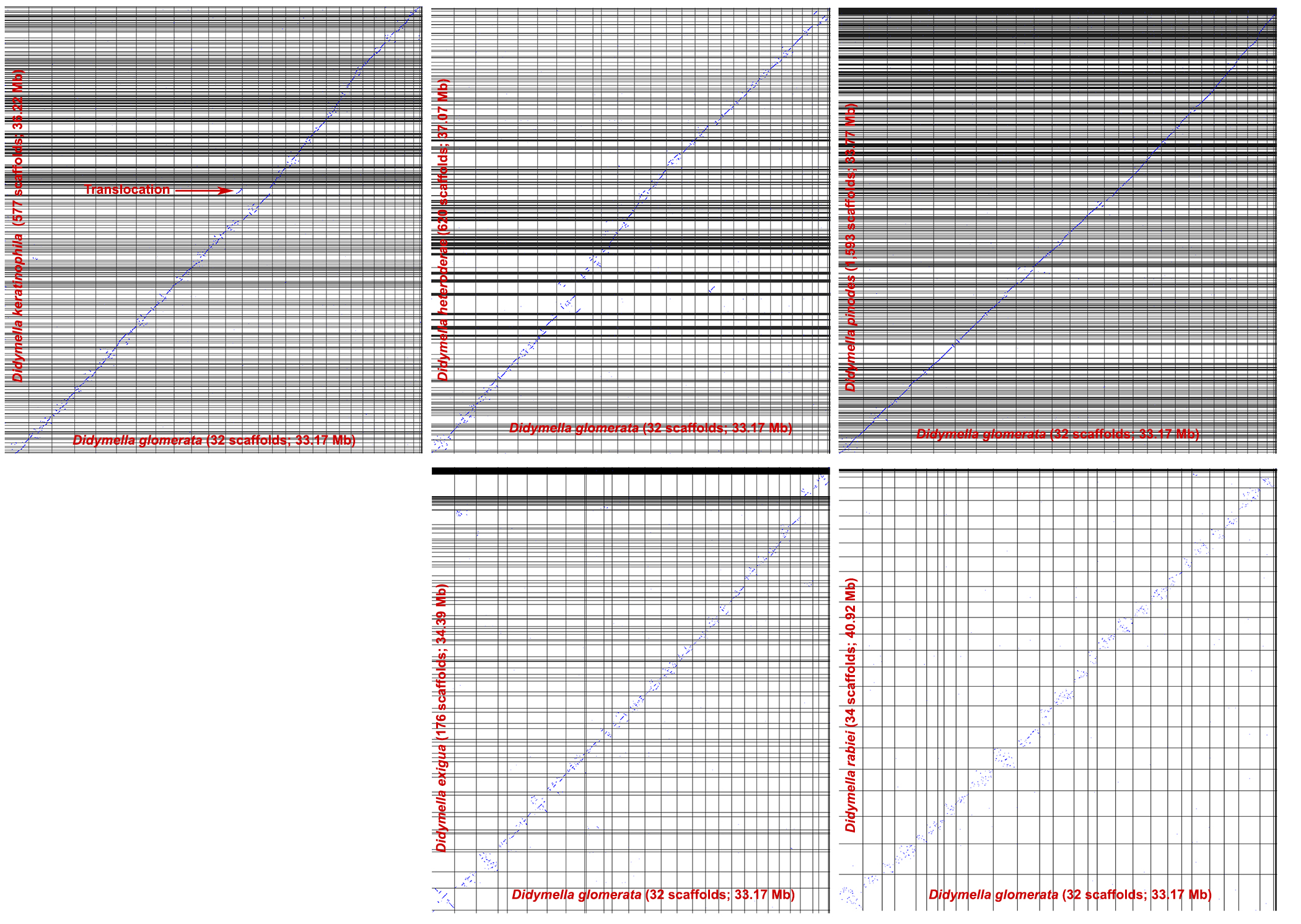

Supplement: Supplementary Figure 1 — Genome collinearity of D. glomerata Pj-2 with Didymella keratinophila 9M1, Didymella heteroderae, Didymella pinnodes WTN-11-157, Didymella exigua CBS 183.55 and the Didymella (Ascochyta) rabiei ArMe14. [file Image_1.jpg]

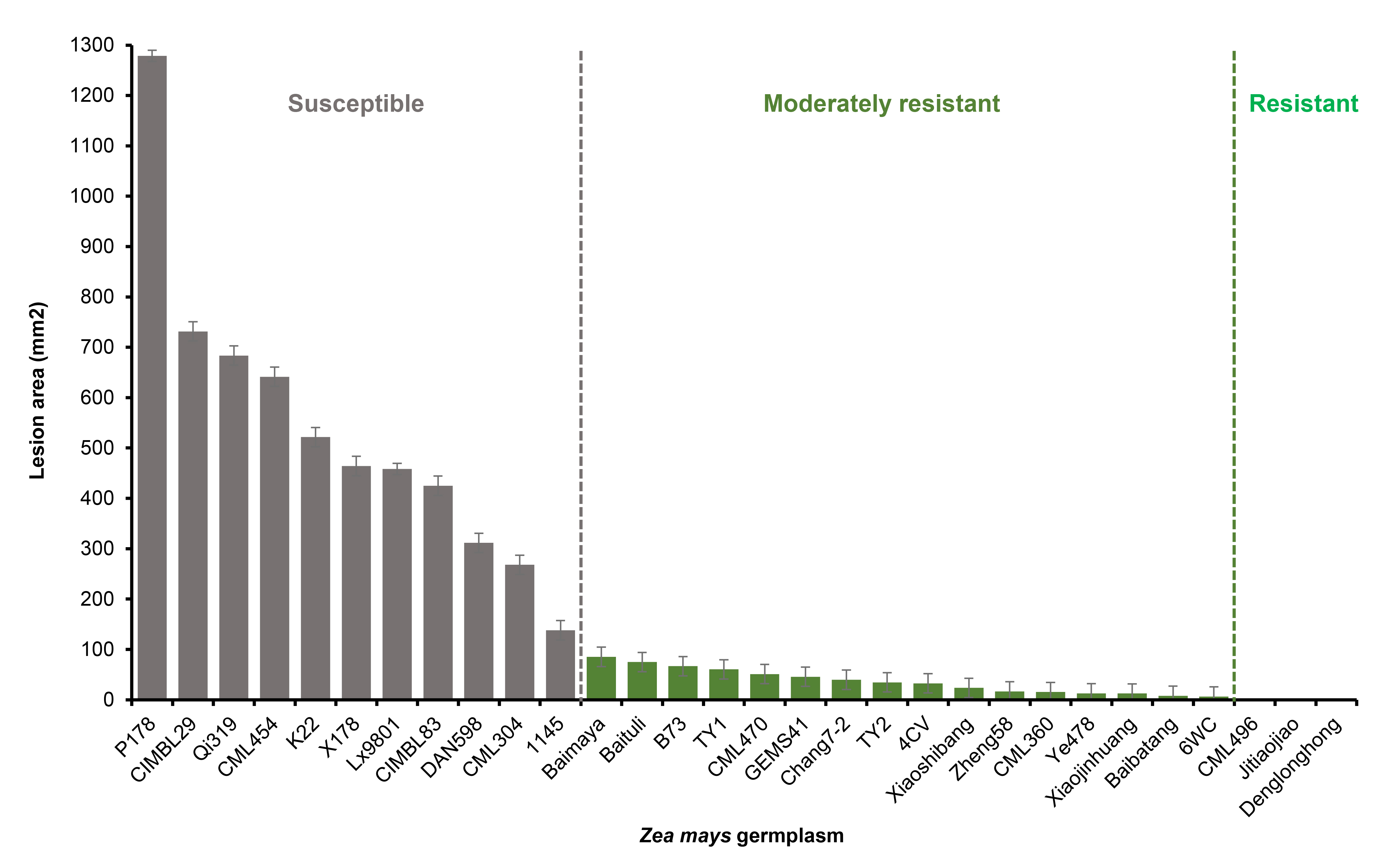

Supplement: Supplementary Figure 2 — Reactions of the Didymella glomerata strain Pj-2 on 30 maize lines representing cultivars, inbred lines and landraces five days post-inoculation. Lesion areas were measured using ImageJ (https://imagej.net/). [file Image_2.jpg]
